# An integrated experimental and analytical approach for the analysis of the mechanical interaction between metal porous scaffolds and bone: implications for stress shielding in orthopedic implants

**DOI:** 10.3389/fbioe.2025.1562367

**Published:** 2025-04-09

**Authors:** Roberto Ramaglia Amadasi, Giulia Rogati, Erica Liverani, Alberto Leardini, Paolo Caravaggi

**Affiliations:** ^1^ IRCCS Istituto Ortopedico Rizzoli, Movement Analysis Laboratory and Functional Evaluation of Prostheses, Bologna, Italy; ^2^ Department of Industrial Engineering, Università di Bologna, Bologna, Italy

**Keywords:** porous scaffolds, bone strain, digital image correlation, stress shielding, finite element analysis, CoCrMo, Ti6Al4V, orthopedics

## Abstract

**Introduction:**

Metal porous structures are becoming a standard design feature of orthopedic implants such as joint endoprostheses. The benefits of the pores are twofold: 1) help improve the cementless primary stabilization of the implant by increasing osteointegration and 2) reduce the overall stiffness of the metal implant thus minimizing stress-shielding. While the mechanical interaction between porous implants and bone has been extensively investigated via complex numerical and finite element models, scarce is the *in vitro* and *in vivo* data on the effect of porosity and materials on stress and strain distribution in the implant-bone compound.

**Materials and methods:**

An integrated numerical and experimental approach was used to investigate the effect of material and porosity on the mechanical interaction in compression between porous metal scaffolds and bovine cortical bone. 18 × 18 × 6 mm cuboid samples were cut from fresh-frozen bovine cortical bones. A 9 × 6 × 6 cavity was obtained in each sample to allow insertion of CoCrMo porous and full density scaffolds. Digital Image Correlation analysis tracked bone strain during axial compression of the scaffold-bone samples up to bone failure. The experimental strain data were compared to those from finite element analysis (FEA) of the scaffold-bone compound. The effect of scaffold porosity and material - Ti6Al4V and CoCrMo - on bone strain distribution and reactions forces, with respect to full bone samples, was assessed via FEA and an analytical spring-based model of the bone-scaffold compound.

**Results:**

The experimental data revealed that the porous scaffold resulted in bone strain closer to that of the intact bone with respect to full density scaffolds. FEA showed that Ti6Al4V scaffolds result in bone strain and reaction forces closer to the those in the intact bone with respect to those in CoCrMo scaffolds. The 1,000 µm pores scaffolds resulted significantly more effective in improving reaction forces with respect to the 500 µm pores scaffolds.

**Conclusion:**

The present findings confirm that metal porous scaffolds help promote a more uniform distribution to the bone compared to full density implants. Ti6Al4V scaffolds demonstrated a more favorable mechanical interaction compared to CoCrMo. This integrated approach offers valuable insights into the design of orthopedic implants with optimized mechanical and osseointegration properties.

## Introduction

Total joint replacement is a widely used surgical intervention to restore function and reduce pain in symptomatic joints following trauma or in case of severe osteoarthritis (Jüni et al., 2006). Population growth, aging, and increased life expectancy have led to a significant rise in total hip and knee replacements and thus of revision surgeries (Kurtz et al., 2007). In the United States, the annual number of primary total hip arthroplasty procedures is projected to increase by 129% by 2030 and 284% by 2040. Similarly, the figures for total knee arthroplasty are expected to rise by 182% and 401%, respectively (Singh et al., 2019). While around 95% of total hip arthroplasties are clinically successful at 10-year follow-up, 15% of patients still require revision surgery. In the first 2 years after surgery, implant failure is primarily due to joint instability and infection; after 5 years, aseptic loosening becomes the leading cause of failure, accounting for 90% of all revision procedures (Ulrich et al., 2008).

A key biomechanical cause of aseptic loosening is the stress shielding (Sundfeldt et al., 2006), caused by a mismatch in mechanical properties between the implant and the surrounding bone. High-stiffness implants, such as those made from surgical stainless steel (316 L), cobalt-chromium-molybdenum (CoCrMo) and titanium (Ti6Al4V) alloys, tend to transfer mechanical loading distally while under-stimulating the bone proximally, which can lead to bone resorption, implant-bone micromovements and eventually to implant failure (Frost, 2004; Huiskes et al., 1992; Nagels et al., 2003). Porous metal structures have become a feasible solution to improve osseointegration and reduce stress shielding in orthopedic implants (Pattanayak et al., 2011; Goriainov et al., 2014; Taniguchi et al., 2016; Song et al., 2023). The development of these structures has been enhanced by continuous advancements in additive manufacturing technologies which allow the production of complex geometries, using also medical grade biomaterials, with optimized mechanical and biological properties (Javaid and Haleem, 2018; Tan et al., 2017). Lattices based on the repetition of unit cells with pore diameters ranging from 300 to 1,000 μm have been shown to provide an optimal environment for bone cell survival and proliferation, regardless of the cell type (Tan et al., 2017; Mahmoud and Elbestawi, 2017; Van Bael et al., 2012). The mechanical and biological characterization of porous metal implants, including studies on cell viability, proliferation and colonization, has been extensively documented for both titanium (Amin Yavari et al., 2013; Kadkhodapour et al., 2015; Yu et al., 2020; Zhang et al., 2022) and cobalt-chrome (Caravaggi et al., 2019; Pagani et al., 2021) scaffolds. However, only a few basic experimental studies have investigated the biomechanical interaction between porous scaffolds and bone (Limmahakhun et al., 2017; Liverani et al., 2021). Strain field analysis via Digital Image Correlation (DIC) has proven to be highly effective to assess the mechanical behavior of bone tissue (Belda et al., 2020; Grassi and Isaksson, 2015; Sztefek et al., 2010) and porous metal scaffolds (Zhang et al., 2019; Pagani et al., 2021) under physiological loading conditions *in vitro*. A porous titanium alloy hip implant has been shown to be more effective at reducing stress shielding compared to a similar-geometry full density implant (Arabnejad et al., 2017). The DIC outcome was further supported by finite element analysis (FEA) (Arabnejad et al., 2017). While FE models are increasingly reliable and accurate in predicting the stress/strain response of bone tissue subjected to physiological loading conditions, their validation and generalization require *in vitro* experimental data (Cristofolini and Viceconti, 1999).

Still, most studies on stress shielding of orthopedic devices rely solely on FEA. A systematic literature review on porous designs for orthopedic joint replacements optimized for stress-shielding reported that 76% of the studies were purely computational, followed by studies combining computational and *in vitro* experiments (15%) and those based solely on *in vitro* experiments (7%) (Safavi et al., 2023).

This study aimed to use an integrated analytical and experimental approach to investigate the influence of porosity and material properties on the mechanical interaction in compression between metal porous scaffolds and bovine cortical bone. The outcome of *in vitro* experimental tests was compared to those from FEA and from a spring-based model of the scaffold-bone compound.

## Material and methods

### Experimental model

#### CoCrMo lattice scaffolds

Porous metal scaffolds were modelled by the repetition of 1,500 µm edge elementary cubic cells, featuring 1,000 µm holes and 1,200 µm spherical cavities (scaffold P1000). The mechanical and biological properties of scaffolds based on this unit cell were previously reported by the same authors (Pagani et al., 2021; Liverani et al., 2021; Caravaggi et al., 2019). 6 × 6 × 9 mm lattice scaffolds were fabricated via Laser Power bed Fusion (MYSINT100, SISMA SpA, Vicenza, Italy) of CoCrMo powder (Liverani et al., 2021) along with a full density scaffold with the same dimensions.

#### Bone-scaffold specimens

Five 18 × 18 × 6 mm cuboid samples were cut (Remet TR60) and milled (proLIGHT) from fresh adult bovine cortical bone specimens. 6 × 6 × 9 mm cavities, matching the metal scaffolds’ dimensions, were precisely milled into four bone samples. To maintain hydration, the bone specimens were wrapped in gauze soaked with physiological-saline and freshly frozen at −20°C. The bone specimens were slowly defrosted at 4°C approximately 24 h before mechanical testing.

**FIGURE 1:**
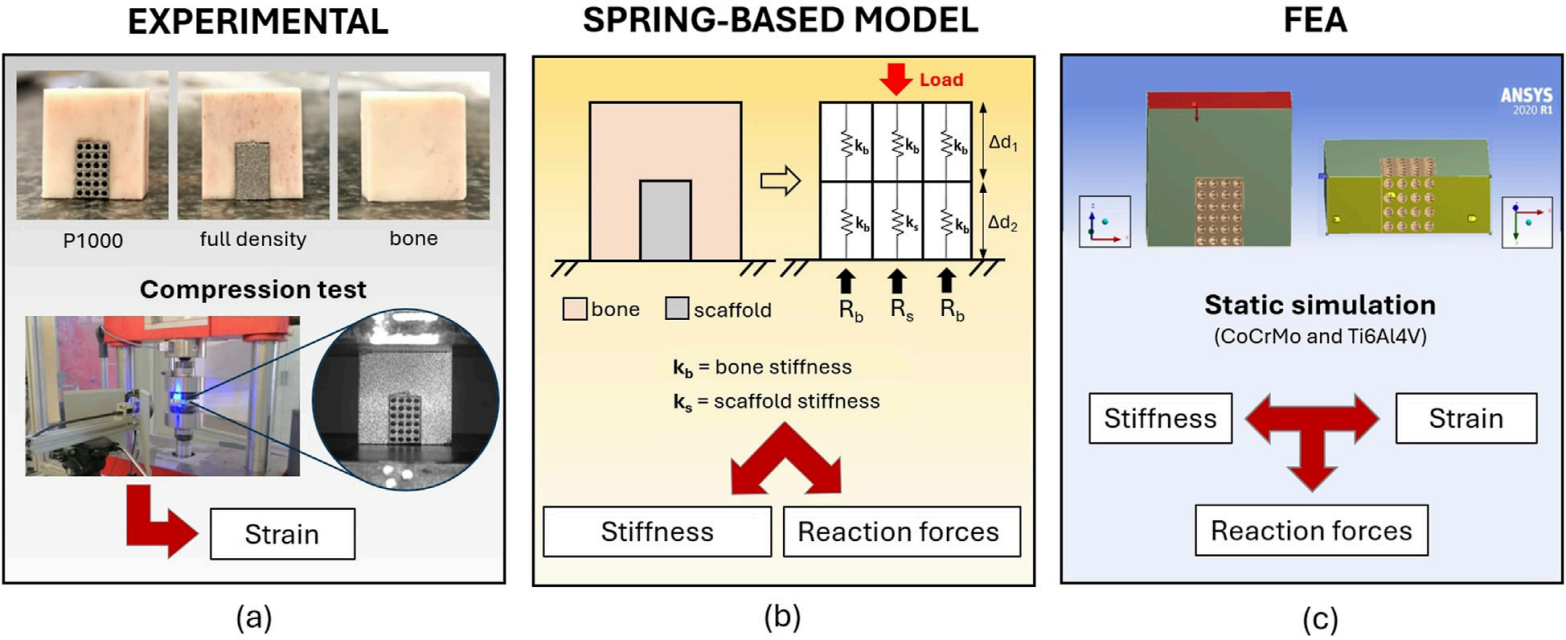
The integrated experimental-analytical approach for the analysis of the mechanical interaction between metal scaffolds and bovine cortical bone. Where: **(a)** is the experimental approach; **(b)** is the analytical spring-based model, and **(c)** is the finite element model.

For experimental compressive tests, bone-implant compounds were prepared by inserting the CoCrMo porous scaffolds into the corresponding cavities of three bone specimens (*P1000* samples, n = 3). One bone specimen was paired with a full density scaffold (*full density* sample, n = 1), while a full bone specimen without cavity was used as control (*bone* sample, n = 1) (Figure 1a).

#### Compression tests

Compression tests were conducted using a uniaxial servo-hydraulic testing machine (Italsigma, Forlì, Italy) equipped with a 20 kN load cell. Tests were performed at room temperature, with the actuator operating under displacement control (0.003 mm/s). The bone-implant compounds were loaded along the longitudinal anatomical direction of the bone, aligned with the z-axis of the testing machine. Force–displacement data (N-mm) were recorded for each specimen undergoing compression until failure.

#### DIC (digital imaging correlation) analysis

**FIGURE 2:**
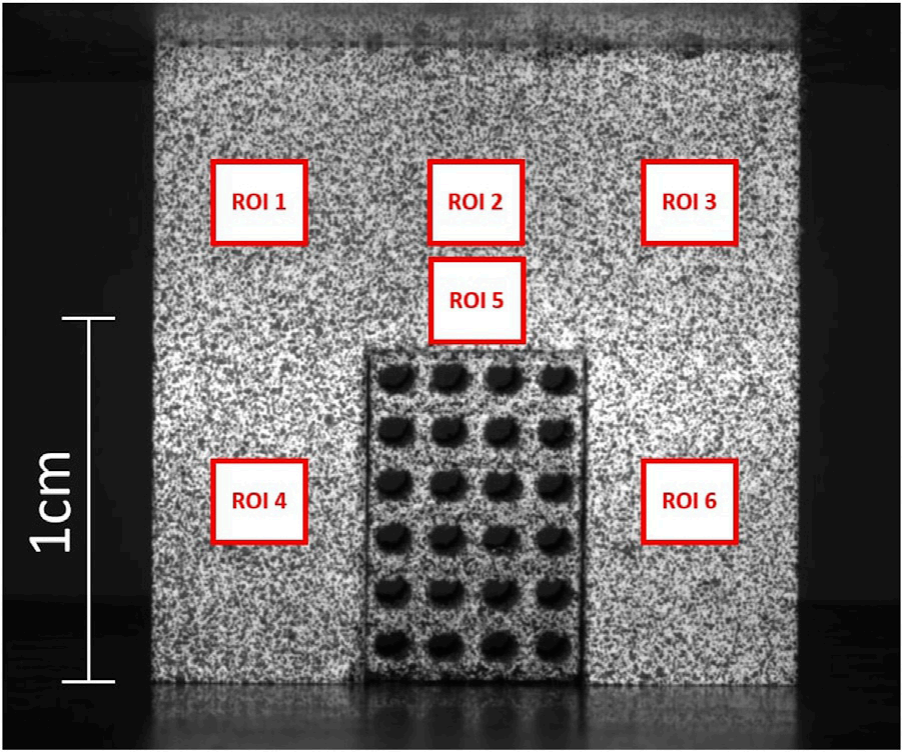
An exemplary DIC image from the experimental tests. A P1000 porous scaffold, fitting a bone cavity of the same dimensions, is subjected to compression. ROIs 1 to 6 are the regions of interest that were used to measure strain distribution in the bone.

During compression tests, a DIC system was implemented to acquire the local strain distribution. White spray paint was used to create a white background on the surface of interest for DIC analysis of each specimen. Subsequently, black spray paint was applied to create a speckle pattern. Still high-resolution images of the bone-implant compounds were taken at 1000 N load increments (6.4 MPx Basler acA3088-57 μm monochrome camera). GOM Correlate (ZEISS Quality suite 4.4) was used to measure local displacements and strains for each pixel identified in the same region of interest (ROI). Six 3 × 3 mm ROIs were identified on the bone portion of each specimen (Figure 2). Ten strain measurements were taken across each ROI and then averaged to characterize the mechanical behavior of the bone in the six ROIs. Repeatability of bone strain data in each of the six ROIs was assessed via Coefficient of Variation (CV), i.e., the standard deviation/sample mean x 100.

#### Spring-based model

The bone-implant compound was ideally divided into six linear-elastic homogeneous cuboid elements, each measuring 6 × 6 × 9 mm, and characterized by springs with different compression stiffnesses for the bone and the CoCrMo scaffold (Figure 1b). The spring-based model was used to estimate the static equilibrium reaction forces at the base of the bone-implant compound under the same boundary conditions as the experimental compressive tests. To test the effect of scaffold porosity on the scaffold-to-bone force distribution, a 500 μm pores CoCrMo lattice scaffold was also modelled (P500 scaffold).

The linear elastic stiffness of both the full density CoCrMo scaffolds and of the bone elements comprising the spring-based model were calculated using Equation 1.

$$k=\frac{E*A}{h}$$ (1)

where: k is the stiffness (kN/mm); E is the Young’s modulus (MPa); A is the cross-sectional area of the element (mm^2^), and h is the height of the element (mm).

The CoCrMo alloys Young’s modulus (199.95 GPa) was taken from the ANSYS Workbench R1 2020 software library. This allowed improving the consistency of the outcome between spring-based and FE models. A Young’s modulus of 8 GPa was used for the bovine cortical bone, as reported in (Liverani et al., 2021).

The stiffness of the unit cells (108 kN/mm and 31 kN/mm for the P1000 and P500, respectively) were determined using Equation 2 from (Liverani and Fortunato, 2021).

$$\frac{1}{k_{tot}}=\sum \frac{{NL}_{Pn}}{NU_{Pn}*k_{U,Pn}}$$ (2)

where: k_tot_ is the total stiffness of the scaffold (N/mm); NL_Pn_ is the number of unit cells’ layers; NU_Pn_ is the number of units per layer, and kU_Pn_ is the stiffness of the unit cell (N/mm).

The solution of a system of four equilibrium equations (Equation 3) allowed for the determination of the reaction forces (Equation 4) at the bone/ground interface (R_b_) and at the scaffold/ground interface (R_s_).

$$\left\{\begin{array}{l} L=3k_{b}*∆d_{1\;}\;\\ L=\left(2k_{b}+k_{s}\right)*∆d_{2}\;\\ L=2R_{b}+R_{s}\;\\ R_{s}=k_{s}*∆d_{2}\;\left(k_{s}\gg k_{b}\right) \end{array}\right.$$ (3)

$$\left\{\begin{array}{l} R_{b}=\frac{L}{2}-\frac{L*k_{s}}{4k_{b}+2k_{s}}\\ R_{s}=\frac{L*k_{s}}{2k_{b}+k_{s}}\; \end{array}\right.$$ (4)

Where: L is the total axial load (N) applied to the upper region; Δd_1_ is the vertical displacement (mm) of the upper region; Δd_2_ is the vertical displacement (mm) of the lower region; k_b_ is the stiffness (N/mm) of the bone elements, k_s_ is the stiffness (N/mm) of the scaffold.

Upper and lower regions were assumed to undergo uniform and independent displacements, exhibiting linear elastic behavior.

#### FE-based model

FEA (Ansys, Workbench R1 2020) was used to estimate stress and strain in the bone and scaffolds, as well as the reaction forces with the ground during static compression, under the same boundary conditions of the experimental tests (Figure 1c). 3D models of the bone-implant compounds were created in Rhinoceros (vers. 6). Material properties of CoCrMo and Ti6Al4V alloy scaffolds were taken from the ANSYS material library. Poisson’s ratio was set to 0.3. A uniformly distributed load up to 10 kN was applied to the top surface of the bone-implant compounds, while the bottom surface nodes were constrained to simulate contact with the ground. The contact between metal scaffolds and the cortical bone was modeled using the ANSYS “no separation” option, which allows for sliding motion while preventing penetration. All simulations were performed under static conditions.

Before running the analyses, the mesh was optimized by reducing the element size until the main simulation outcomes converged. Quadratic hexahedral elements were used for the bone and the bone-implant compound fitted with full density metal scaffolds, while quadratic tetrahedral elements were used for those fitted with the porous metal scaffolds. Six ROIs were identified by selecting the relevant mesh nodes to allow comparison with the experimental DIC strain data in the same ROIs. The average strains in each ROI were calculated for each simulation.

## Results

### Experimental model

**FIGURE 3:**
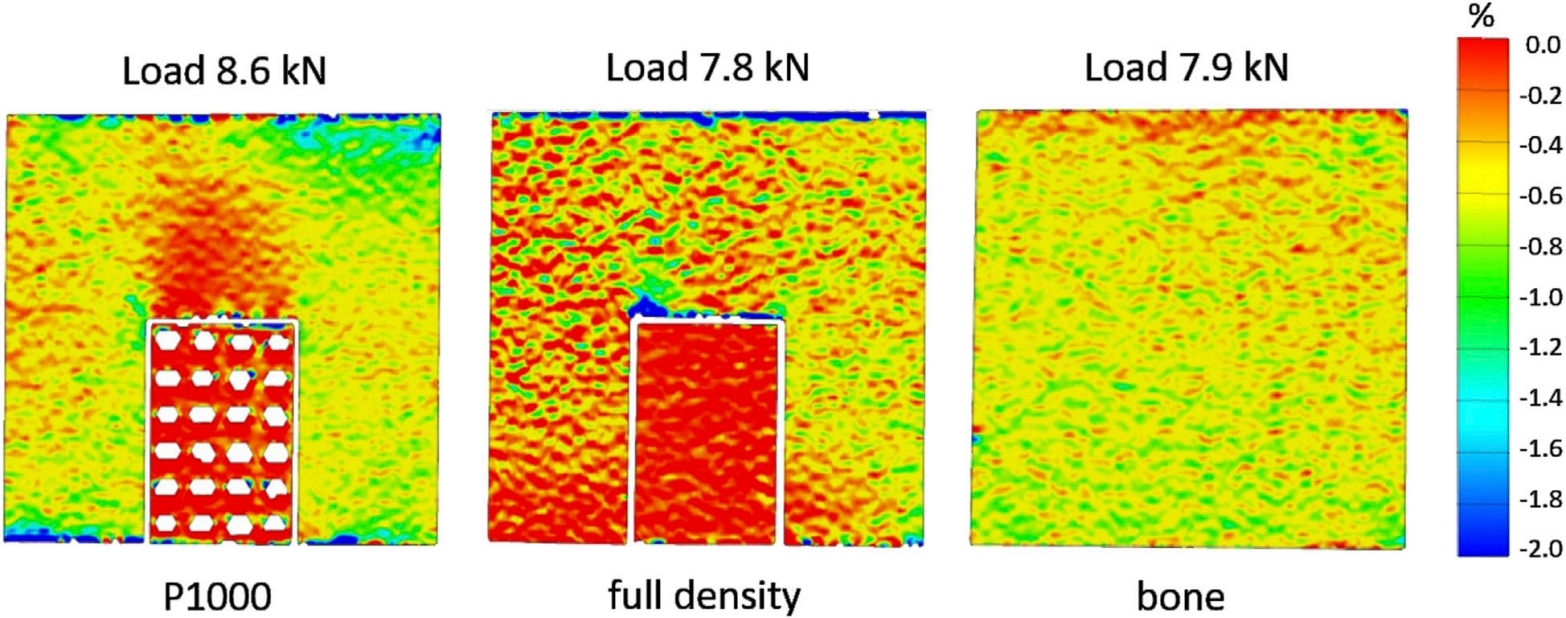
DIC strain maps in the P1000, full density and bone specimens under compression.

**FIGURE 4:**
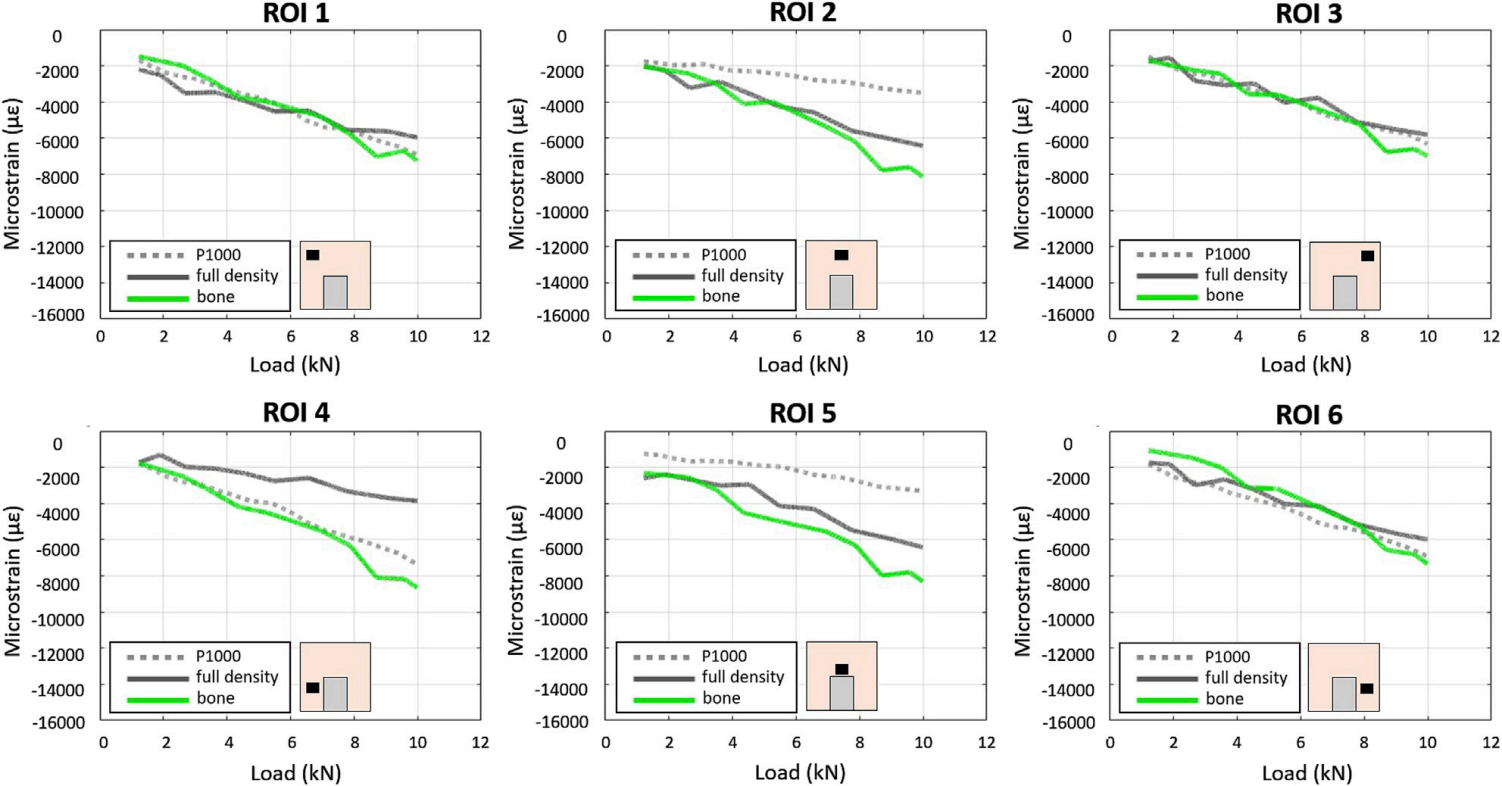
Experimental–DIC based - load/strain relationships in the 6 ROIs in the P1000, full density and bone specimens under compression.

As far as the compression of the full bone specimen, DIC analysis (Figures 3, 4) showed uniform strain distribution on the sample surface (≈7,500 µε).

In the P1000 samples (bone + porous scaffold), about the same max strain (≈7,000 µε) was observed in ROIs 4 and 6 (lateral bottom surfaces) and ROIs 1 and 3 (lateral upper surfaces). ROIs 2 and 5 (central upper region) showed the lowest strain (≈3,500 µε) (Figure 4).

In the full density sample, the max strain was similar across all ROIs (≈6,000 µε) except for ROI 4, which showed relatively lower strain (≈4,000 µε).

The largest differences (≈4,200 µε) between the P1000 and the full bone specimens were observed in the central regions (ROIs 2 and 5), while the largest difference (≈4,000 µε) between the full density and the full bone specimens was observed in ROI 4.

At 5 kN compression, the mean CV of bone strain data for the three P1000 samples across the six ROIs was 19% (range 3%–56%).

### Spring-based model

The estimated stiffness of the CoCrMo scaffolds calculated according to (Liverani and Fortunato, 2021) is reported in Table 1. As expected, scaffold porosity was inversely correlated with scaffold stiffness. The stiffness of the full density scaffold was approximately 9 times greater than that of the P1000 scaffold, which was closer to the stiffness of the bone (32 kN/mm).

**TABLE 1:** The first two columns report the FE-based estimation of stiffness for the P1000, P500 and full density metal scaffolds in Ti and CoCrMo alloys. The third column shows the stiffness of the CoCrMo scaffolds estimated *via* Equation 1 (full density scaffolds and bone) and Equation 2 (porous scaffolds).

	FE-based stiffness [kN/mm]		Analytical stiffness [kN/mm]
	Ti6Al4V	CoCrMo	CoCrMo
P1000 scaffold	68.5	119.9	82.7
P500 scaffold	190.4	333.5	288.0
Full density scaffold	427.9	800.3	799.8
Cortical bovine bone	32.0		32.0

By replacing k_s_ and k_b_ in Equation 4 with the estimated stiffness above, the reaction forces at the base of the bone-implant compound were calculated. Table 2 is reporting the % variation of reaction forces at the base of the bone portion of the bone-implant compound with respect to those calculated for the full bone specimen used as control. The lowest variations were observed for the P1000 samples.

**TABLE 2:** Changes in reaction force [%] at the bone/ground interface for P500, P1000 and full density CoCrMo and Ti alloys scaffolds under compression, with respect to that in the full bone (no scaffold). The changes were estimated *via* FEA (first two columns) and using the analytical spring-based model (last column).

	Δ reaction force FE-based model		Δ reaction force Spring-based model
	Ti6Al4V	CoCrMo	CoCrMo
Full density scaffold	−41.2%	−44.1%	−88.9%
P500 scaffold	−34.4%	−39.6%	−72.7%
P1000 scaffold	−18.2%	−27.7%	−34.6%

### FE-based model

**FIGURE 5:**
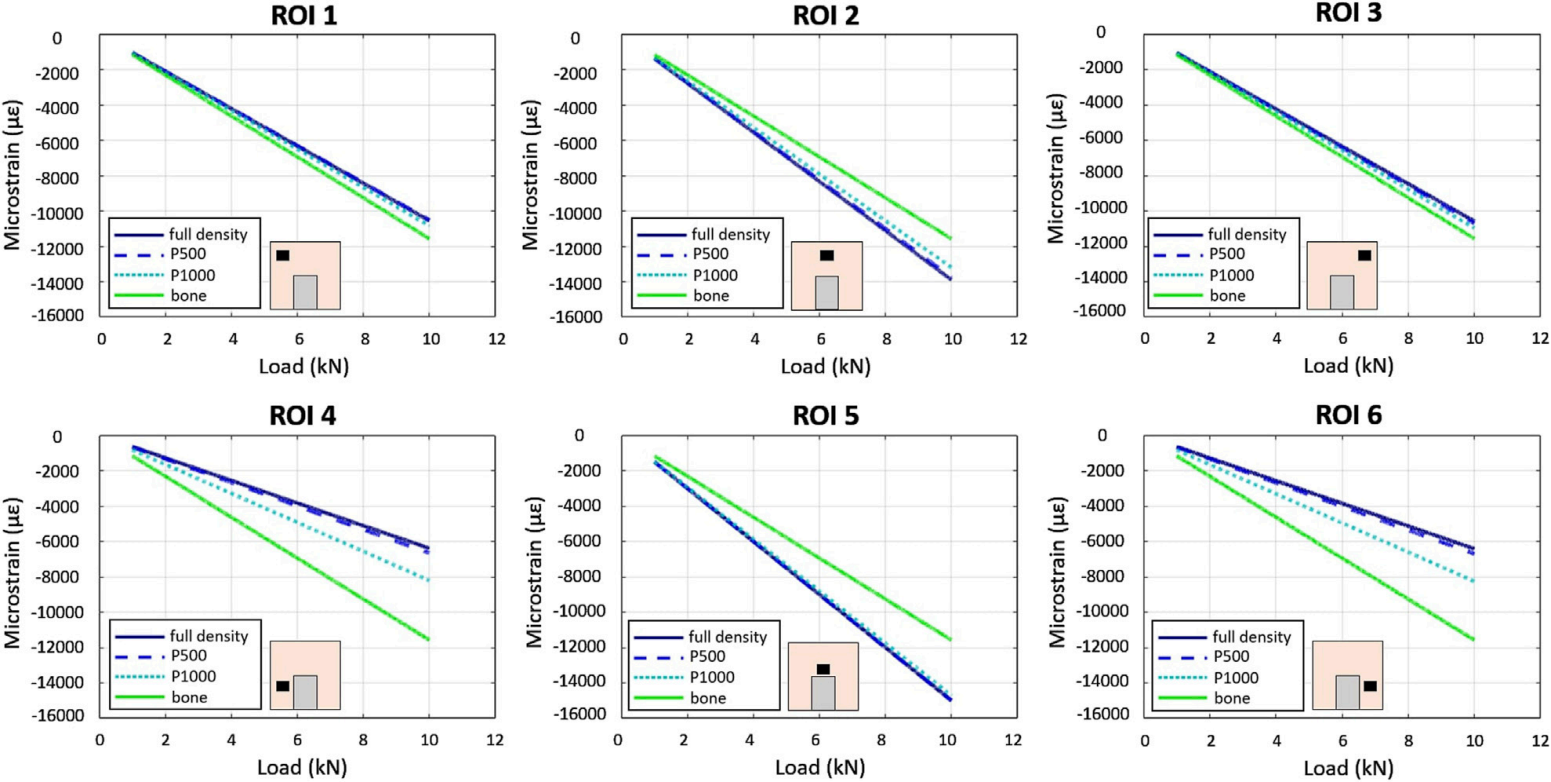
In-silico–FE based - load/strain relationships in the 6 ROIs in the P1000, P500, full density and bone specimens under compression. Scaffolds’ material is CoCrMo.

Linear relationships between compressive force and strain were observed across all specimens and in all ROIs using the FEA model (Figure 5).

In the full bone specimen, strain was uniformly distributed across all ROIs. In the CoCrMo full density, P500, and P1000 specimens, ROIs 2 and 5 exhibited the highest strain, while significantly lower strains were observed in the bone region adjacent to the implant (i.e., ROIs 4 and 6). Strains in the CoCrMo P1000 specimen were similar to those observed in the full bone specimen.

**FIGURE 6:**
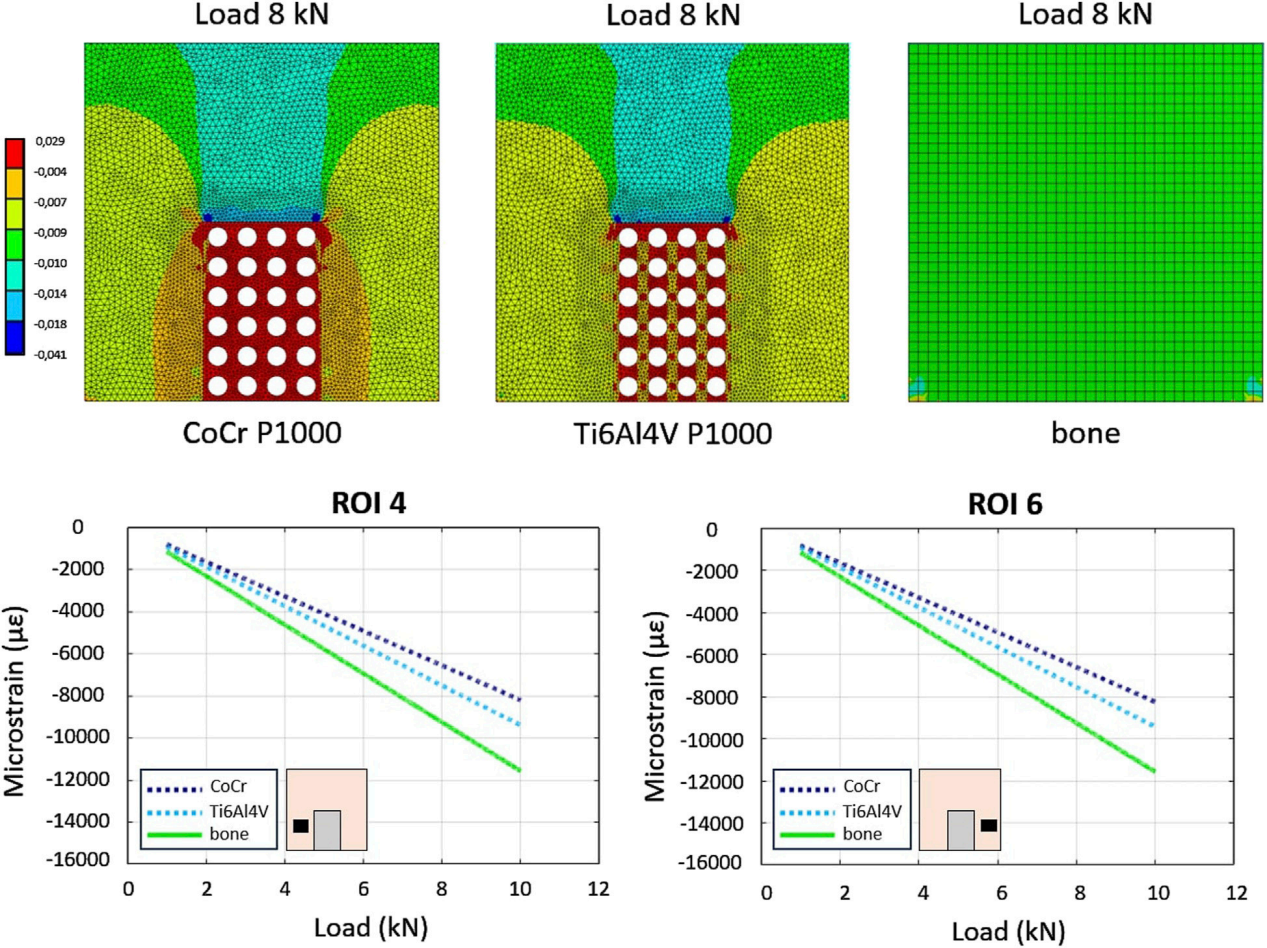
*In-silico*–FE-based - comparison between load-strain relationships in the P1000 specimen between CoCrMo and Ti alloys. The bone sample is used as control.

As far as the simulated compression of the Ti6Al4V alloy bone-implant compound, the average strains in the six ROIs of the full density and P500 specimens were comparable. The P1000 specimen exhibited bone strains which were more closely resembling the control configuration (i.e., full bone specimen) especially in ROIs 4 and 6 (Figure 6).

As observed in the spring-based model, greater scaffold porosity resulted in reaction forces more similar to those of the full bone specimen (Table 2). In addition, the Ti6Al4V alloy scaffolds showed smaller differences in reaction forces with respect to the CoCrMo scaffolds.

## Discussion

Joint prostheses are designed to restore joint function and mobility, promoting rapid, controlled healing and long-term integration with surrounding tissues (Grzeskowiak et al., 2020; Puleo and Nanci, 1999). However, a mismatch in stiffness between implants and bone can lead to stress shielding, a phenomenon that alters load distribution and results in bone resorption in underloaded regions. Mechanical strain, independent of macro-displacement, is a key stimulus for bone formation, regeneration, or resorption (Frost, 2004). While dynamic loading significantly influences bone remodeling (Ozcivici et al., 2010), the biomechanical interaction between orthopedic devices and bone tissue is still not fully understood. This study aimed to better elucidate this interaction through an integrated experimental and analytical approach.

**FIGURE 7:**
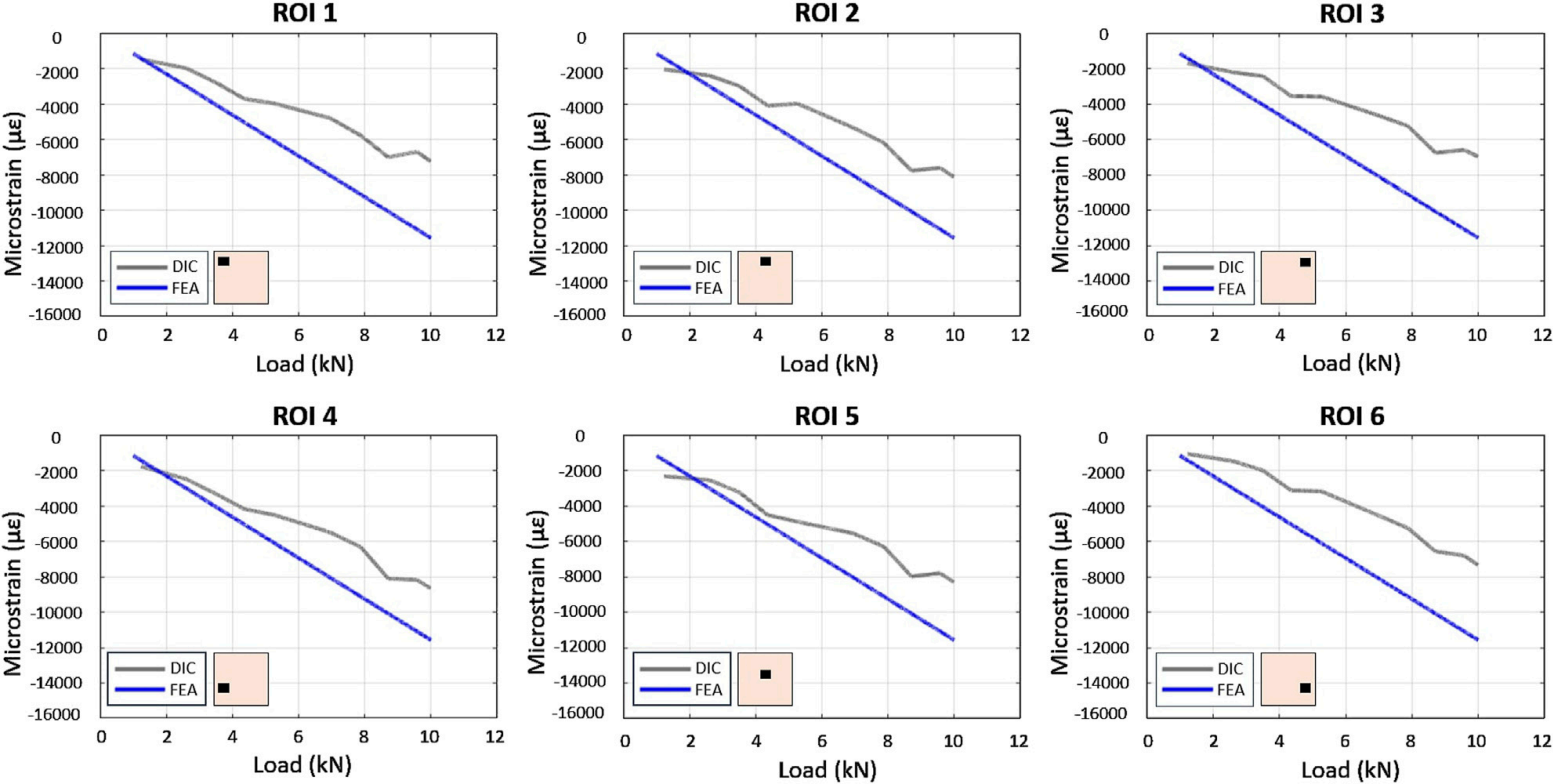
Comparison between FE and experimental load/strain relationships in the 6 ROIs in the bone specimen under compression.

In the experimental model, DIC analysis quantified strain fields at the bone-implant interface under compressive loading. Both FEA and experimental models predicted a homogeneous strain distribution when the full bone specimen was loaded. However, DIC strains were slightly lower than the FEA estimates (Figure 7). The introduction of metal implants significantly altered strain distribution. Both DIC and FEA revealed symmetrical strain patterns and reduced strain in lower peri-implant regions with highly porous CoCrMo scaffolds. However, FEA predicted higher strains in the upper regions compared to DIC. This discrepancy may be attributed to incomplete contact between the scaffold and the bone in the central region, potentially due to scaffold surface roughness. While less pronounced, a similar pattern was observed with the full density implant.

The comparison of CoCrMo scaffold stiffness and reaction forces between FE and spring-based models showed consistency. As expected, porosity inversely correlated with scaffold stiffness. FE- and spring-based models predicted a 6-fold and 9-fold reduction in stiffness, respectively, when transitioning from full density to P1000 scaffolds (Table 1). This reduced stiffness resulted in more physiological reaction forces at the bone-implant interface with porous scaffolds (Table 2). Optimizing implant design requires minimizing regions of abnormal stress, as these can induce bone resorption and implant loosening (Frost, 2004). Among the analyzed designs, the porous P1000 scaffold emerged as the most suitable for achieving uniform load distribution and minimizing stress concentrations. Additionally, scaffold porosity enhances osseointegration by facilitating bone ingrowth, contributing to secondary implant stability and long-term success (Goriainov et al., 2014).

FEA of Ti6Al4V P1000 scaffolds revealed bone strains similar to those of the full bone specimen (Figure 6). Ti6Al4V scaffolds exhibited similar stiffness-porosity relationships as CoCrMo scaffolds (Table 1), but overall lower stiffness due to material properties. Consequently, reaction forces at the bone base were more similar to those of the intact bone in Ti6Al4V scaffolds (Table 2).

While the present experimental model of the mechanical interaction between porous implant and bone appears to be more consistent with the *in vivo* implantation of orthopedic devices, thus improving upon the model reported in (Liverani et al., 2021), the outcome of the study should be interpreted considering some limitations. These include the limited number of experimental samples and the potential for incomplete contact, despite careful fitting, between the scaffold and bone in the experimental model. This may have contributed to discrepancies between FE and experimental strain data, especially in the upper central bone region. However, despite the limited sample size, the repeatability of DIC-based bone strain data can be acceptable for such experimental tests. The spring-based model’s assumptions of linear elasticity, homogeneity, and constant cross-sectional area, along with the neglect of the bone’s viscous and anisotropic properties, may have influenced the results. Additionally, the difference in loading conditions between FE (force control) and experimental (displacement control) tests should be considered.

The multi-method approach used in this study to analyze the mechanical interaction between bone and metal scaffolds in compression provides evidence that highly porous metal scaffolds can promote a more uniform load transfer to the surrounding bone compared to full density implants. This effect is more evident when Ti6Al4V alloy is used as scaffold’s material. While the analysis of multi-axial and dynamic loading conditions, more consistent with *in vivo* physiological conditions, should be sought in future endeavors, this study provides novel quantitative information on the mechanical interaction between metal devices and bone. This data may be applied to develop orthopedic devices with optimized osseointegration properties and reduced stress-shielding.

## Data Availability

The raw data supporting the conclusions of this article will be made available by the authors, without undue reservation.
